# Uterine cavity assessment in infertile women: Sensitivity and specificity of three-dimensional Hysterosonography versus Hysteroscopy

**Published:** 2013-12

**Authors:** Firoozeh Ahmadi, Zohreh Rashidy, Hadieh Haghighi, Mohamadreza Akhoond, Maryam Niknejadi, Mandana Hemat, Mansour ShamsiPour

**Affiliations:** 1 *Department of Reproductive Imaging at Reproductive Biomedicine Research Center, Royan Institute for Reproductive Biomedicine, ACECR, Tehran, Iran.*; 2 *Department of Statistics, Mathematical Science and Computer Faculty, Shahid Chamran University, Ahvaz, Iran.*; 3 *Department of Endocrinology and Female Infertility at Reproductive Biomedicine Research Center, Royan Institute for Reproductive Biomedicine, ACECR, Tehran, Iran.*; 4 *Department of Epidemiology and Reproductive Health at Reproductive Epidemiology Research Center, Royan Institute for Reproductive Biomedicine, ACECR, Tehran, Iran.*

**Keywords:** *Three-dimensional hysterosonography*, *Hysteroscopy*, *Uterine cavity*

## Abstract

**Background:** Assessment of uterine abnormalities is a core part in infertility evaluation.

**Objective: **The aim of this study was to evaluate the sensitivity and specificity of three-dimensional hysterosonography (3-DHS) in the diagnosis of uterine abnormalities in infertile women.

**Materials and Methods:** The infertile women who visited Royan Institute and referred to 3-DHS consecutively, prior to in vitro fertilization, from 2010-2011 included in this cross-sectional study. For patients who underwent hysteroscopy in addition to 3-DHS (214/977), the verification bias adjusted sensitivity and specificity of 3-DHS which were calculated by global sensitivity analysis method. Hysteroscopy was used as the gold standard for diagnosis of uterine abnormalities. Histological diagnosis of resected endometrial tissues by hysteroscopy was assessed and the adjusted sensitivity and specificity of 3-DHS and hysteroscopy in detection of polyp or hyperplasia were determined. Histopathologic results were considered as the gold standard for diagnosis of polyp or hyperplasia.

**Results:** The overall sensitivity and specificity for 3-DHS in diagnosis of uterine anomalies considering hysteroscopy as the gold standard were 68.4% and 96.3% respectively. Sensitivity and specificity of hysteroscopy in diagnose of polyp or hyperplasia was calculated at 91.3% and 81.4% respectively. Sensitivity and specificity of 3-DHS in diagnosis polyps or hyperplasia was calculated at 91.4% and 80.2 % respectively.

**Conclusion:** The results of present study proved that, compared to hysteroscopy; 3-DHS has a reliable specificity for diagnosis of uterine abnormalities. Sensitivity and specificity of 3-DHS and hysteroscopy in detecting polyp or hyperplasia regarding histopathology as the gold standard was the same.

## Introduction

Uterine abnormalities can be one of the etiologies of infertility by interfering with implantation (1). Assessment of uterine abnormalities is a core part in infertility evaluation. A variety of modalities such as hysterosalpingography (HSG), transvaginal sonography (TVS), diagnostic hysteroscopy, two dimensional hysterosonography (2-DHS) and three dimensional hysterosonography (3-DHS) can be used for the diagnosis of uterine abnormalities (1, 2). However diagnostic hysteroscopy has remained the gold standard in infertility investigation (3-5). The validity and limitation of every method were measured and discussed in published data. 

While TVS as an initial investigation is unable to differentiate intrauterine pathology with complete certainty, 3-DHS as a noninvasive procedure may be recommended as a proper alternative for diagnostic hysteroscopy for diagnosis of uterine abnormalities in infertile women with less costs and complications (6, 7). The primary aim of our study was to determine the sensitivity and specificity of 3-DHS versus diagnostic hysteroscopy in the evaluation of uterine abnormalities in infertile women, and secondly to compare sensitivity and specificity of 3-DHS and hysteroscopy in diagnosis of polyp or hyperplasia regarding histopathology as the gold standard. We hope this study can be helpful in management of uterine abnormalities from reproductive perspective.

## Materials and methods

In this cross-sectional study, a total of 977 infertile women referred to 3-DHS consecutively, prior to in vitro fertilization; from 2010-2011 at Royan Institute were included. Two hundred fourteen patients underwent hysteroscopy in addition to 3-DHS. The mean age of study group was 33.3±3.83 SD (24-47) years. The time interval between 3-DHS followed by hysteroscopy was not longer than three months. Both hysteroscopy and 3-DHS examinations were performed 7-10 days from the start of menstruation. Hysteroscopy was used as the gold standard because it was previously reported (3-5). 3-DHS images were interpreted by one of two experienced radiologists (more than 10 years’ experience) with a special training in gynecology. Hysteroscopy was performed by an experienced gynecologist. Throughout this study, two patients were excluded due to cervical stenosis and failure of catheter insertion. 3-DHS was performed using three-dimensional extended imaging (3DXI) (ACCUVIX XQ, Medison, South Korea) ultrasound with a 6.5-MHz transvaginal probe. We used a balloon-tipped silicone urine Foley’s catheter (NO. 6 Supa. Tehran, Iran). 

After a baseline TVS, catheter was advanced through the cervical canal and into the lower uterine segment. The transvaginal transducer was reinserted and followed by the instillation of 5-20 ml sterile saline through the catheter under direct ultrasound guidance until an adequate distention of the uterine cavity. Representative transverse and longitudinal images were obtained. In this study 3DXI was employed as a new display modality. This study was approved by research ethics committee and institutional review board at Royan Institute and informed written consent was obtained from the patients.

Diagnosis by 3-DHS was recorded as follows: endometrial polyp or hyperplasia (hyperechoic thickening of the endometrial mucosa); intrauterine adhesions (filmy, mobile band traversing the uterine walls); submucous liomyoma (solid, whorled, mixed echogenic tumor, which can be disrupts and affects the endometrial interfaces). In this study size and location of polyps or hyperplasia, myoma and length of septum were documented by simultaneous display and outlining the region-of-interest (ROI) in three perpendicular planes. The measurements of submucousal uterine myoma were performed according to the Europian Society of Hysteroscopy considering the degree of myometrial extention (completely within the cavity, with ≥50% protruding into cavity and with <50% endocavitry projection) (8). Mullerian duct anomalies were recorded based on the classification of American Fertility Society (AFS). 

We reported length of septum and also the relative percentage of uterine fundus to the distance between two ostiuma in the case of the septated uterus. Histological diagnoses of resected endometrial tissues were available in 142 patients. And the pathologic diagnoses of these specimens were compared with hysteroscopic and 3-DHS findings, and the sensitivity and specificity of each test were calculated regarding histopathology as the gold standard. In 46/142 cases endometrial tissue specimens were obtained by curettage during hysteroscopy and in 96/142 cases were obtained by hysteroscopy-guided biopsy. Hysteroscopy was performed by experienced gynecologists. In this study distention of the cavity was obtained using normal saline or sorbitol. 

The hysteroscopy was performed under either general or regional anesthesia using a Storz 4mm hysteroscope (Karl. Storz-GmbH and Co. Tuttlingen, Germany) by an expert gynecologist. For the calculation of sensitivity and specificity all patients were examined by both the diagnostic test (3-DHS) and the gold standard test (hysteroscopy). A total of 977 patients who referred for 3-DHS included within this study but from them only 214 patients underwent hysteroscopy. From 214 patients who underwent hysteroscopy histological diagnosis of only 142 patients were available. 

All patients who underwent 3-DHS were not referred to an invasive gold standard due to ethic reason and, the results of all patients was not verified by the gold standard test and patients with positive test on 3-DHS had more likely to do hysteroscopy and histopathology but patients with negative test on 3-DHS had less chance to do the gold standard (hysteroscopy or histopathology) and as a consequence the sample of patients that verified by the gold standard test was not representative of the whole patients and calculation of the sensitivity and specificity based on this sample is bias. This type of bias is called verification or work-up bias (9, 10).

In recent years many methods have been proposed to adjust sensitivity and specificity for verification bias. 

**Statistical analysis**

In this article we used global sensitivity analysis to estimate the verification bias adjusted sensitivity and specificity of 3-DHS with respect to hysteroscopy and histopathology (95% confidence intervals) (11, 12). In addition to statistical analysis, data management and data documentation performed by SPSS Statistics version 16.0.

## Results

**Figure 1:**
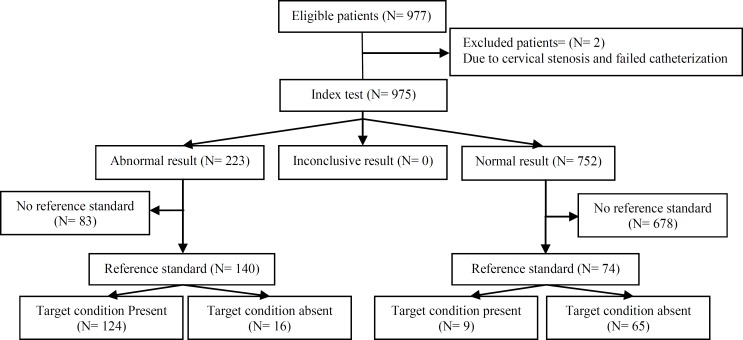
Results for the diagnosis of normal and abnormal cases of uterine pathologies diagnosed by hysteroscopy and 3-DHS

The duration of couples’ infertility ranged from 1-16 years. Among the 977 women, 223 (22.87%) had abnormal test results, and 752 (77.12%) had normal test results on 3-DHS (Figure 1). Results for the diagnosis of normal and abnormal cases of uterine pathologies diagnosed by hysteroscopy and 3-DHS were illustrated in table I. As shown in table I, the 3-DHS had 68.4% sensitivity and 96.3% specificity for the diagnosis of uterine abnormalities regarding hysteroscopy as the gold standard. 

The hysteroscopy diagnosed 133 cases of uterine abnormalities. The 3-DHS was in complete agreement in 124 of 133 cases. The number of patients with more than one uterine abnormality were 9. The hysteroscopy revealed 96 polyps or hyperplasia, 10 liomyoma, 6 synechiae, 27 septated uterus and 1 unicornuate uterus. The 3-DHS was in complete agreement in 87/96 cases of polyps or hyperplasia, 7/10 cases of liomyomas, 5/6 cases of synechiae, 22/27 cases of septated uterus and 1/1unicornuate uterus and 65/74 cases of normal uteruses. The sensitivity, specificity of 3-DHS versus hysteroscopy in diagnosing uterine abnormality was shown in table II.

Histological diagnosis of resected endometrial tissues was available in 142 patients, 136 polyps and 6 myomas were revealed by histopathology. Histological report was not available in 61 normal cases and 11 cases of polyps (due to scanty tissues or patient refusal to take tissue to the laboratory for testing). 3-DHS in the detection of endometrial polyp’s hyperplasia showed 91.4% sensitivity and 80.2% specificity (Table III). Detection of endometrial polyps or hyperplasia by hysteroscopy had sensitivity of 91.3%, specificity of 81.4% (Table IV). 

In 22 cases endometrial polyps or hyperplasia were diagnosed by both 3-DHS and hysteroscopy which was not confirmed by histopathology. In these 22 cases histopathology reported a normal endometrium (mostly proliferative phase of endometrium). Both Hysteroscopy and 3-DHS were failed to detect 3 cases of polyps or hyperplasia which was revealed by histopathology (Table III and IV) .

**Table I:** Verification bias adjusted sensitivity and specificity of three-dimensional hysterosonography (3-DHS) versus hysteroscopy in diagnosing congenital and acquired uterine abnormality

	**Hysteroscopy (+)**	**Hysteroscopy (-)**	**Unverified**	**Total**	**Sensitivity% (95%C.I.)**	**Specificity% (95%C.I.)**
3-DHS (+)	124	16	83	223	68.4 (54.4,0.82.4)	96.3 (94.5,98.1)
3-DHS (-)	9	65	678	752		
Total	133	81	761	977		

CI: confidence interval

**Table II:** The sensitivity and specificity of 3-DHS versus hysteroscopy in diagnosing uterine abnormality: (arcuate or septate uterus and unicornuate uterus) and acquired uterine pathologies (Hyperplasia or polyp, myomas and adhesion)

**Uterine lesion**	**Sensitivity% ** **(95% C.I.)**	**Specificity% ** **(95% C.I.)**
Polyp or hyperplasia	65.9 (50.8, 81.0)	98.8 (97.9,99.7 )
Submucosal myoma	53.8 ) 26.5, 8.11(	99.7) 99.2,100)
Synechiae	63.8 (14.5, 100)	99.8 (99.5, 100)
Septated uterus	75.3 (53.1, 97.5)	98.9 (98.0, 99.8)

**Table III:** Verification bias adjusted sensitivity and specificity measures of 3-DHS in the diagnosis of endometrial polyp or hyperplasia, regarding histopathology as the gold standard

	**Histopathology** ** (+)**	**Histopathology** ** (-)**	**Unverified**	**Total**	**Sensitivity% (95%C.I.)**	**Specificity% (95%C.I.)**
3-DHS (+)	62	23	11	96	91.4 (82.3,100)	80.2 (72.9, 87.5)
3-DHS (-)	3	48	61	112		
Total	65	71	72	208		

**Table IV:** Verification bias adjusted sensitivity and specificity measures of hysteroscopy in the diagnosis of endometrial polyp or hyperplasia, regarding histopathology as the gold standard

	**Histopathology** ** (+)**	**Histopathology** ** (-)**	**Unverified**	**Total**	**Sensitivity (95%C.I.)**	**Specificity (95%C.I.)**
Hysteroscopy (+)	62	22	10	94	91.3 (82.1,100)	81.4 (74.3, 88.5)
Hysteroscopy (-)	3	49	62	114		
Total	65	71	72	208		

**Table V:** Parameter of diagnostic accuracy in relative studies

**Author study**	**Test**	**Reference**	**Sample size**	**Sensitivity%**	**Specificity%**	**NPV%**	**PPV%**
Sylvestre et al (4)	3-DHS	Hysteroscopy	59	100	-	-	92
Alaetebi F et al (14)	3-DHS	Hysteroscopy	100	100	100	100	100
Diaferia D et al (15)	2-DHS	Hysteroscopy	91	98	93	-	-
Markris N et al (16)	3-DHS	Hysteroscopy	121	91	98	96	97
Guven MA et al (17)	2-DHS	Hysteroscopy and/or Histopathology	93	90	40	77	65
Acholonu Jr. UC et al (18)	2-DHS	Hysteroscopy	93	81.8%	93.8%	-	-
Ayida G et al (19)	2-DHS	Hysteroscopy	38	87.5	100	100	91.6
Aboulghar MM et al (20)	3-DHS	Hysteroscopy	77	100	100	86.4	92.4

NPV: negative predictive value

PPV: positive predictive value

## Discussion

The acquired and congenital uterine anomalies are important causes of female infertility, hence, investigation of the uterine cavity is obligatory practice in the assessment of infertile women particularly prior to assisted reproductive technology (ART) cycles (1). The non-invasive ultrasound imaging as a diagnostic triage tool continues to improve management of infertile patients. De Kroon CD *et al* reported that diagnostic hysteroscopy can be limited to or inconclusive hysterosonography and it was able to replace 84% of the outpatient diagnostic hysteroscopies (13). The number of studies comparing 3-DHS and hysteroscopy in infertile population are few. However, regarding the table V these studies concluded that hysterosonography is an accurate method in detection of endometrial cavity abnormalities with sensitivity and specificity of more than 80% and 40%, respectively (4, 14-20). Since it is often unethical or impractical to verify all study patients (by referring all of them for hysteroscopy), retrospective adjustments are needed to provide correct inferences about the accuracy of tests.

In this study we aim to minimize effect of verification bias, therefore, the point estimate sensitivity of 3-DHS was relatively low (68.4%). However the specificity (96.3%) was in agreement with previous studies (15, 16, 18). Therefore, 3-DHS as a highly specific test will be most helpful for clinician when the test result is positive and obviates the need for further investigation of abnormal findings. The verification bias is a common problem when two or more continuous tests are used in sequence. In two review studies 26% and 33% of published articles were related to the accuracy of tests which was reported to have verification bias but failed to recognize it (21, 22). Philbrick *et al* reviewed 33 studies on the accuracy of exercise tests for coronary disease and found that 31 might have had verification bias (23). The verification bias can distort the estimated accuracy of a diagnostic test and may lead to two approaches. In one method estimation of sensitivity and specificity is done only based on all patients who underwent both gold standard and diagnostic tests. 

This method is called naive approach and cause sensitivity grossly overestimated and specificity underestimated. In the second approach for patients who did not verified by the gold standard test the result of diagnostic test were considered as the same as the gold standard. In this approach, the sensitivity and specificity grossly are overestimated. The interesting finding in our study was the same false positive rate (22 cases) for hysteroscopy and 3-DHS in the diagnosis of polyp or hyperplasia. In this study histopathologic samples were obtained either by guided biopsy (96/142) or curettage (46/142), whereas emerging evidence from recent literature suggests that blind hysteroscopic biopsy or curettage has low diagnostic accuracy. This may be explained by the fact that a small polyp or hyperplasia were probably missed by curettage or may be crushed during curettage and can yield scant tissue, insufficient for diagnosis (17, 24-26). 

Guven *et al* have reported a high false-positive rate (22/61) 36% for hysterosonography in the diagnosis of polyps. Of which (14/22) 64% was due to a thick endometrium in secretory phase mimicking a polyp in hysterosonography (17). We reduced this possibility by performing 3-DHS during the 7-10^th^ days of the menstruation cycle when the endometrium is thin and this can also be achieved by reconstructing 3D images in order to get a more precise representation of the intracavitery pathologies. It is important to note the limitations which were encountered during this study. 

In this study pathologic correlation was not available for all subjects, even when intracavitary abnormalities were reported on 3-DHS images. Some patients were reluctant to send the specimens to lab due to financial issues. The number of potential technical difficulties exists in performance of 3-DHS. Difficulty of the catheter placement and vague images rarely was occurred during the procedure. Although the use of balloon catheter improves the optimal distention of uterine cavity, still in a few cases sub optimal distention affects the quality of images.

## Conclusion

We minimize sources of verification bias which can greatly affect assessment of the diagnostic utility of 3-DHS. According to our results, compared to hysteroscopy; 3-DHS has a reliable specificity for diagnosis of uterine abnormalities and it can be introduced as a first line investigation tool in an infertility work up (27-28). Sensitivity and specificity of 3-DHS and hysteroscopy in detecting polyp or hyperplasia regarding histopathology as the gold was the same.

## Conflict of interest

There is no conflict of interest or financial interests that might inappropriately influence or interfere with research findings. There was no financial aid to support this study**.**
